# Prevalence & features of inner ear malformations among children with congenital sensorineural hearing loss: A Public Cochlear Implant Centre Experience

**DOI:** 10.12669/pjms.36.7.3134

**Published:** 2020

**Authors:** Jawwad Ahmed, Ghulam Saqulain, Muhammad Iqbal Javed Khan, Mobeen Kausar

**Affiliations:** 1 Dr. Jawwad Ahmed, FCPS (Otolaryngology), Associate Surgeon, Department of Otolaryngology & Cochlear Implantation, Capital Hospital PGMI, Islamabad, Pakistan; 2 Dr. Ghulam Saqulain, FCPS (Otorhinolaryngology), Head of Department of Otorhinolaryngology & Cochlear Implantation, Capital Hospital PGMI, CDA, Islamabad, Pakistan; 3 Dr. Muhammad Iqbal Javed Khan, FRCS. Consultant Otologist & Skull Base Surgeon, Department of Otorhinolaryngology, Bradford Teaching Hospitals NHS Foundation Trust, England; 4 Dr. Mobeen Kausar, MPH. Deputy Medical Superintendent, Healthcare Commission Coordinator, DHQ Hospital, Rawalpindi, Pakistan

**Keywords:** Computed tomography scan, Middle ear malformations, Prevalence

## Abstract

**Objective:**

To determine the prevalence and features of inner ear anomalies in children with congenital profound hearing loss who presented at our cochlear implant center based on imaging studies.

**Methods:**

This retrospective study reviewed charts of children with congenital SNHL, who presented to Department of Otolaryngology & Auditory Implant Centre, Capital Hospital Islamabad over a period of 2 years from 1^st^ May 2017 to 30^th^ April 2019. These included 481 cases of both genders aged between 1 to 12 years. After gathering demographic data, audiological data, computed tomography findings of the temporal bone were analyzed. Data was analyzed using SPSS 22.

**Results:**

The Inner Ear Malformations were identified in 48(10%) children including 28 (58.33%) males and 20 (41.67%) female. Most 20(41.67%) presented at >3-5 years of age followed by 19(39.58%) at 2-3 years. However, no significant association of gender (p=0.57, p=0.076) and age of presentation (p=0.344, p=0.697) for right and left ears was noted with inner ear malformations. The most common anomaly noted were CLA, CH-III and CH-II in decreasing order of frequency in both ears.

**Conclusion:**

The prevalence of IEM’s was found to be 48(10%). Commonest anomalies noted were CLA, CH-III and CH-II. No significant association of gender and age of presentation was noted with type of anomaly in both ears.

## INTRODUCTION

Cochlear implant services for the deaf children got established in Pakistan over the last two decades, however no local studies report the prevalence of inner ear malformations. This is important in planning surgical treatment and predicting prognosis.

With a local prevalence of congenital anomalies of 7%, around 11.94% suffer from malformations of face, neck, eyes and ears.^1^ Inner ear anomalies are usually encountered while diagnosing and operating pediatric cases with non-serviceable hearing and both membranous and bony malformations may be encountered.^2^ Joshi VM et al, has reported a high prevalence of IEMs (15 to 20%) in cases with severe or profound hearing loss (HL)^3^ with fear of intra-operative complications. Hence identification of bony anomalies is important in planning cochlear implants (CI) surgery.^4^ In spite of the fact that hearing and speech development do not differ in post implant cases with and without IEMs^5^, however more difficulties may be faced with IEM’s including gusher and facial nerve abnormalities.^6^

Evaluation of cochlear implant candidates requires imaging studies in addition to audiological investigations to delineate anomalies of the temporal bone and membranous structures like 8^th^ cranial nerve (CN), inner ear (IE), middle ear (ME) and outer ear (OE). High-resolution computed tomography (CT) scan and Magnetic resonance imaging (MRI) studies are gold standard imaging studies required in such cases and used in conjunction with each other.^7^ Among other structures, a High-resolution CT can help visualize coexisting ME and OE malformations and anatomic variants, while MRI has more sensitivity for delineating 8^th^ CN and spaces in the IE filled with perilymph and endolymph and brain anomalies.^3^

A variety of congenital anomalies are seen both syndromic and non-syndromic^7^, with varying presentation, radiological and audiological features and present with different findings on surgical intervention.^2^ These anomalies have been grouped into eight groups by Bajin SL^2^, including Complete aplasia of labyrinth; Rudimentary otocyst; Cochlear aplasia; Common cavity; Cochlear hypoplasia; Incomplete partition-I, Incomplete partition-II, Incomplete partition-III; Enlarged vestibular aqueduct; and Cochlear aperture abnormalities. The bony labyrinth originates from mesoderm and therefore its malformations are noted as deficient coils and branching of tubular structures derived from otocyst. Also there may be aplasia or dysplasia of sensory epithelium, and deficient cartilaginous and bony framework.

Frequency of congenital HL in Pakistan has been reported by Ahmed S et al. as 13 per 1000 with 15 % profound and 85% moderate to severe HL, which is quite high compared to other parts of the world^8^, with the first cochlear implantation in a case of IEM in Pakistan reported in 2013.^9^

The richness of HL gene heterogeneity in Pakistani population makes it quite attractive for research^10^, combined with higher prevalence of congenital HL, and establishing of cochlear implant facility in the public sector, prompted and facilitated this study to determine the prevalence and features of inner ear anomalies in children with congenital profound hearing loss who presented at out cochlear implant center based on imaging studies. With this subject lacking in local literature in Pakistan, this study is of immense importance keeping in view high prevalence of HL in Pakistan. Also this could be helpful in providing reliable statistical data regarding IEM’s in patients with congenital HL and will be helpful to plan effective treatment strategies and for research purposes.

## METHODS

This retrospective study reviewed medical charts of children with Sensorineural hearing loss (SNHL), who presented to or were referred to Department of Otolaryngology & Cochlear implant Centre, Capital Hospital, Islamabad, over a period of 2 years from 1st May 2017 to 30th April 2019. These included N=481 cases of both genders aged between one to 12 years. After gathering demographic data, audiological data, computed tomography findings of the temporal bone were analyzed. Study was conducted after obtaining ethical approval of the research from Ethical Review Committee of Capital Hospital PGMI, Islamabad vide Ref No. 2029-05-002 dated 7th May, 2020.

After collection, data was tabulation using Microsoft Excel Worksheet and analyzed statistically using Statistical Package for Social Studies (SPSS) Version-23. Results were analyzed using descriptive statistic including frequencies, percentages, mean, and standard deviation. Chi-square test was used to see gender and age association. The data was then viewed along with national and international literature and resulting deductions were discussed.

## RESULTS

Current study included 286(59.5%) males and 195(40.5%) females with a mean age of 3.62 ± 2.29 years. The frequency of middle ear anomalies was 48(10%). Of the 48 Right Ears, 5(10.4%) were normal, the commonest anomaly was CLA 9(18.8%), followed by CH-III 8(16.7%) and CH-II 5(10.4%) (Table-I). Of the 48 Left Ears, 7(14.6%) were normal and commonest anomaly noted was CLA & CH-III 8(16.7%) each, followed by CH-II in 6(12.5%) cases. Individual anatomic deformities noted are summarized in Table-II.

**Table-I:** Anomaly type * Gender & Age Group Association. Cross Tabulation (n=48).

Ear	Anomaly Type n(%)	Gender Association			Age Group Association				

		F(20)	M(28)	X2, P-Value	<2.00 (6)	2.00-3.00 (19)	>3.00-5.00 (20)	>5.00-12.00 (3)	X2, P-Value
Right Ear Anomalies	Normal 5(10.4)	2	3	0	4	1	0	38.82, .344
	VCN-1 2(4.2)	0	2	1	1	0	0	
	VCN-2b 1(2.1)	1	0	0	0	1	0	
	CLA 9(18.8)	3	6	1	2	5	1	
	LSCC Anomaly 4(8.3)	0	4	0	2	2	0	
	CH-I 4(8.3)	2	2	2	1	0	1	
	CH-II 5(10.4)	2	3	0	4	1	0	
	CH-III 8(16.7)	5	3	0	3	4	1	
	CH-IV 1(2.1)	1	0	1	0	0	0	
	SCC Anomaly 3(6.3)	2	1	1	1	1	0	
	Ext Ear Anomaly 1(2.1)	0	1	0	0	1	0	
	IP-II Anomaly 3(6.3)	1	2	0	0	3	0	
	CA 2(4.35)	1	1	0	1	1	0	
Left Ear Anomalies	Normal 7(14.6)	4	3	19.55 .076	1	4	1	1	31.18 0.697
	VCN 2(4.2)	0	2		1	1	0	0	
	VCN 1b 1(2.1)	0	1		0	0	1	0	
	CLA 8(16.7)	0	8		1	2	5	0	
	LSCC Anomaly 2(4.2)	0	2		0	1	1	0	
	CH-I 3(6.3)	2	1		1	1	0	1	
	CH-II 6(12.5)	2	4		0	5	1	0	
	CH-III 8(16.7)	6	2		0	3	4	1	
	CH-IV 2(4.2)	1	1		1	0	1	0	
	SCC Anomaly 5(10.4)	3	2		1	2	2	0	
	VCN 3 1(2.1)	0	1		0	0	1	0	
	IP-II Anomaly 2(4.2)	2	0		0	0	2	0	
	Ext Ear Anomaly 1(2.1)	0	1		0	0	1	0	

**Table-II:** Individual congenital anatomic deformities * Ear. Cross tabulation. (N=48).

Congenital Anatomic Deformity		Ear	

Area	Anomaly	Right n (%)	Left n (%)
VIII N	Normal	27(56.3)	29(60.4)
	Absent	16(33.3)	14(29.2)
	Thin	5(10.4)	5(10.4)
IAC	Normal	25(52.1)	28(58.3)
	Reduced	13(27.1)	9(18.8)
	Absent	10(20.8)	11(22.9)
LSCC	Normal	12(25)	17(35.4)
	Absent	10(20.8)	12(25)
	Small Hypoplastic	26(54.2)	19(39.6)
PSCC	Normal	23(47.9.)	25(52.1)
	Absent	17(37.4)	15(31.3)
	Small Hypoplastic	8(16.7)	8(16.7)
SSCC	Normal	25(52.1)	28(58.3)
	Absent	18(37.5)	16(33.3)
	Small Hypoplastic	5(10.4)	4(8.3)
Cochlea	Normal	16(33.3)	19(39.6)
	Absent	13(27.1)	9(18.8)
	Sac	12(25)	12(25)
	Apical turn absent	5(10.4)	4(8.3)
	Only basal turn present	2(4.2)	4(8.3)

As regards the different IEM’s noted in in the current study, out of the 48 Right Ears, the commonest anomaly being CLA 9(18.8%), followed by CH-III 8(16.7%) and CH-II 5(10.4%). While on the left side, 7(14.6%) were normal ears and the commonest anomaly noted was CLA & CH-III 8(16.7%) each, followed by CH-II in 6(12.5%) cases. As regards individual anatomic deformities noted, 8^th^ Nerve was absent in 16(33.3%) right ears and 14(29.2%) left ears while it was thin in 5(10.4%) of both right and left ears. Internal Auditory Canal (IAC) was reduced in size in 13(27.1%) & 9(18.8%) and absent in 10(20.8%) & 11(22.9%) of Right and Left ears respectively. Lateral Semi Circular Canal was absent in 10(20.8%) & 12(25%); and small hypo-plastic in 26(54.2%) & 19(39.6%) of Right and Left ears respectively. PSCC was absent in 17(37.4%) & 15(31.3%) of right and left ears; and small hypo-plastic in 8(16.7%) of both ears each. SSCC was absent in 18(37.5%) & 16(33.3%); and small hypo-plastic in 4(10.4%) & 4 (8.3%) of Right and left ears each. Cochlea was absent in 13(27.1%) & 9(18.8%); apical turn was absent in 5(10.4%) &498.3%) and only basal turn was present in 2(4.2%) & 4(8.3%) of right and left ears each. While cochlea was just a Sac in 12(25%) of both ears.

There was no significant association of type of anomaly with gender in both right and left ear with p=0.57 & p=0.076 respectively. Also no association of type of anomaly was noted for age with p=0.344 & p=0.697 in right and left ear respectively.

## DISCUSSION

Current study with 286(59.5%) males and 195(40.5%) females, did not reveal any significant association of type of anomaly with gender. In contrast, Zanon A et al. in their review noted that Gender difference with regards to balance, hearing, speech and IEM’s exist, however studies regarding gender prevalence of different malformations are lacking 11, with the exception that high prevalence of outer ear anomalies in males.^12^

With a mean age of 3.62 + 2.29 years and an age range of 1 to 12 years, the majority of IEM (n=20) were reported in age group >3 to 5 years, followed by n=19 in age group 2-3 years, however there was no significant age association with IEM. In contrast Masuda S et al. reported that the prevalence of malformations was significantly (p<0.01) higher in infants (84.6%) compared to those 1 to 15 years age (55.8%).^13^

We noted a high frequency of IEM’s 48/481(10%) in cases with bilateral SNHL. While, a Saudi study by Aldhefeeri & Alsanosi reported a slightly lower prevalence of 7.5%.^14^ International literature shows a highly variable prevalence of IEM’s. In sheer contrast to our study, Masuda and Usui in 2019 reported a high prevalence of 24.3% in cases with Bilateral SNHL, while they noted a low prevalence of 3.7% in cases Unilateral SNHL.^15^ Also a Chinese study by Sun B et al.^16^ reported an incidence of 30.69%. In contrast, Agarwal SK et al. reported a frequency of 13.93%, indicating that probably population in this subcontinent was less prone to develop IEM’s.^17^

Large variations in the results of different studies have been reported. In a Saudi study by Aldhefeeri & Alsanosi Large vestibular aqueduct was commonest pathology (33.3%), followed by dysplasia of SCC (29.1%) and cochlear hypoplasia (4.1%).^14^While in another study of Chinese origin, by Sun B et al. revealed malformations of cochlea (31%), simple vestibular aqueduct (40.33%), and simple vestibular aqueduct/SCC/IAC in 7.35%. Of the malformations of the cochlea Michel deformity was noted in 1.13%, cochlea aplasia in 1.81%, common cavity deformity in 3.17%, incomplete partition type I in 8.62%, cochlea hypoplasia in 9.07% and incomplete partition type II was reported to be present in 76.19%.^16^ While in an Indian study by Agarwal SK et al. the prevalence of cochlear anomaly was 73%, 87.1% had vestibular malformations, 56.4% had vestibular aqueduct malformation, 30.7% were with IAC anomaly and 29.4% had 8^th^ CN anomalies.^17^ In an imaging study by El Sheikh E et al. cochlear hypoplasia the commonest (4.5%), followed by common cavity (3%), IP-1 (3%), IP-2 (16.7%), IP-3 (7.6%), posterior rotated cochlea (6.1%), dilated vestibule (3%), isolated SCC hypoplasia (3.03%), SCC hypoplasia with common cavity (3.03%), dysplastic SCC (6.8%), dilated IAC (4.5%), hypo-plastic IAC (18.2%), VCN hypoplasia (18.2%), isolated EVA (36.4%).^18^ Also in a study by Dhanasingh A, found normal anatomy in four, enlarged vestibular aqueduct in 3, cochlear aplasia in 2, IP-I in 8, IP-II in 3, IP-III in four, CH in 17, common cavity (CC) in 5. Majority of CH cases had cochlear height shorter than four mm whereas the CC cases measured cochlear height above 6 mm. For all the other malformation types, cochlear height was between 4 and 6 mm.^19^

Also in another study, 32(46.4%) had stenosis of 8^th^ nerve canal out of which 13 had canal stenosis alone. While IAC anomalies were seen in 22(31.8%), cochlear anomalies in 14(20.3%) and vestibular and SCC anomalies in 5(7.2%), enlarged vestibular aqueduct in 2(2.9%). The prevalence of narrowed internal auditory canal was significantly more in cases with 8^th^ nerve canal stenosis.^13^ Yi JS et al. reported that 57% cases of unilateral SNHL had 8^th^ nerve canal atresia or stenosis associated with inner ear malformations.^20^ Shama SAM et al. reported that aplasia commonly involves cochlea (26.9%), and semicircular canals (19.23%), while dysplasia commonly involves Vestibule.^21^

## CONCLUSIONS

**Fig.1:**
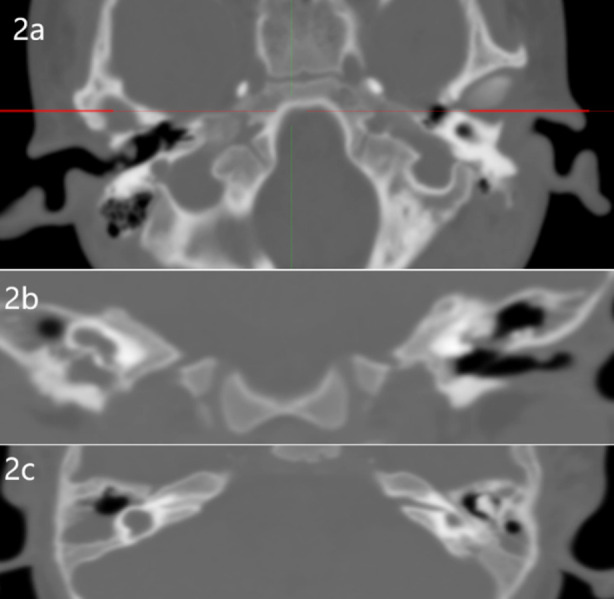
Computed Tomography Scan Axial view showing a) Congenital absence of inner ear & Vth Cranial Nerve bilaterally consistent with CLA, b & c) Hypoplasia of basal turns with 6mm on right side while 4 mm on left side with a cyst like structure replacing Semicircular canals consistent with CH 3.

The prevalence of IEM’s of 48/481 (10%) in the current study was not very high. Commonest anomaly noted were CLA (fig.1a), CH-III (Fig.1b,c) and CH-II. No significant association of gender and age at presentation noted with type of anomaly in both ears

### Authors Contribution

**JA:** Did the data collection, analysis and interpretation of results.

**GS:** Was responsible for writing of manuscript and critical revision and was responsible for integrity of research.

**IJK:** Conceived the idea and critical revision of the manuscript.

**MK:** Did the literature review.
